# Application of neurite orientation dispersion and density imaging (NODDI) to a tau pathology model of Alzheimer's disease

**DOI:** 10.1016/j.neuroimage.2015.10.043

**Published:** 2016-01-15

**Authors:** N. Colgan, B. Siow, J.M. O'Callaghan, I.F. Harrison, J.A. Wells, H.E. Holmes, O. Ismail, S. Richardson, D.C. Alexander, E.C. Collins, E.M. Fisher, R. Johnson, A.J. Schwarz, Z. Ahmed, M.J. O'Neill, T.K. Murray, H. Zhang, M.F. Lythgoe

**Affiliations:** a UCL Centre for Advanced Biomedical Imaging , Division of Medicine, University College London, UK; b Department of Computer Science & Centre for Medical Image Computing, University College London, UK; c Eli Lilly & Co. Ltd, Erl Wood Manor, Windlesham, Surrey GU20 6PH, UK; d Eli Lilly & Co. Ltd, Lilly Corporate Center, Indianapolis, IN 46285, USA; e Department of Neurodegenerative Disease, UCL Institute of Neurology, Queen Square London, UK; f Department of Medical Physics and Bioengineering, Saolta University Health Care Group, University Hospital Galway, Newcastle Road, Galway, H91 YR71, Ireland

## Abstract

Increased hyperphosphorylated tau and the formation of intracellular neurofibrillary tangles are associated with the loss of neurons and cognitive decline in Alzheimer's disease, and related neurodegenerative conditions. We applied two diffusion models, diffusion tensor imaging (DTI) and neurite orientation dispersion and density imaging (NODDI), to *in vivo* diffusion magnetic resonance images (dMRI) of a mouse model of human tauopathy (rTg4510) at 8.5 months of age. In grey matter regions with the highest degree of tau burden, microstructural indices provided by both NODDI and DTI discriminated the rTg4510 (TG) animals from wild type (WT) controls; however only the neurite density index (NDI) (the volume fraction that comprises axons or dendrites) from the NODDI model correlated with the histological measurements of the levels of hyperphosphorylated tau protein. Reductions in diffusion directionality were observed when implementing both models in the white matter region of the corpus callosum, with lower fractional anisotropy (DTI) and higher orientation dispersion (NODDI) observed in the TG animals. In comparison to DTI, histological measures of tau pathology were more closely correlated with NODDI parameters in this region. This *in vivo* dMRI study demonstrates that NODDI identifies potential tissue sources contributing to DTI indices and NODDI may provide greater specificity to pathology in Alzheimer's disease.

## Highlights

• We analyzed the microstructural changes in rTg4510 and wild type mice at 8.5 months.
• We correlated microstructural findings with histological measures of tau burden
• We compare two diffusion MR models: DTI and NODDI.
• Both models revealed changes in tissue microstructure due to tau pathology.
• The NODDI metrics demonstrated a good correlation with histological measures of tau burden.

## Introduction

The progression of soluble tau protein to neurofibrillary tangles (NFTs) is at the centre of many human neurodegenerative diseases, including Alzheimer's disease (AD) (Spillantini and Goedert, 2013). A known function of tau protein is to stabilise axonal microtubules. However, in AD these proteins disassociate resulting in a breakdown of the microtubules and aggregation of insoluble hyperphosphorylated tau filaments, impairing neural pathways (Ballatore et al., 2007). Tau aggregates form within discrete neurites in select nerve cells and propagate, effecting downstream synaptically connected brain regions (de Calignon et al., 2012, Pooler et al., 2013, Ahmed et al., 2014). Monitoring the progression of tau and its effect on neuronal reorganization due to tau-induced neurodegeneration *in vivo* is a key requirement in understanding the progression of AD and determining the efficacy of current therapeutic attempts to target tauopathies (de Calignon et al., 2012, Ahmed et al., 2014).

To study these effects, we used an animal model of tau pathology: the TG mouse model which overexpresses a mutant form of human tau (P301L) resulting in tau accumulation in the form of NFTs largely restricted to the hippocampus, cortex, olfactory bulb, and striatum (Santacruz et al., 2005). These NFTs are an intracellular hallmark of AD and are believed to lead to neuronal dysfunction, neurotoxicity and brain atrophy resulting in neurological deficits and neuronal cell death (Santacruz et al., 2005). Current understanding of tau propagation in synaptically connected brain regions is primarily obtained from invasive tissue measurements using immunohistological methods (Liu et al., 2012, Hardy and Revesz, 2012), which are restricted to single time point analyses and do not allow *in vivo* dynamic assessment of tissue remodelling due to pathology.

Diffusion magnetic resonance imaging (dMRI) is sensitive to the diffusion process of MR visible molecules. In biological tissue, dMRI techniques provide indices associated with the dispersion pattern of water molecules, which reflect the integrity of the tissue microstructure *in vivo* (Basser and Pierpaoli, 1996). Most previous studies of AD related pathologies have applied a tensor model referred to as diffusion tensor imaging (DTI) to investigate changes in neuronal cytoarchitecture (Assaf and Pasternak, 2008, Horsfield and Jones, 2002) and age related changes in the TG mouse model (Sahara et al., 2014, Wells et al., in press). However, the common diffusion tensor model is based on the assumption of a simple underlying Gaussian diffusion process (Alexander et al., 2002). In contrast, the neurite orientation dispersion and density imaging (NODDI) technique uses a non-Gaussian biophysical model, providing higher sensitivity to the non-monoexponential diffusion in the brain parenchymal environment (Alexander et al., 2002, Syková and Nicholson, 2008). In tauopathies, where the densely packed microtubules in neurite structures become destabilised and tau protein misfolds to form NFTs, diffusion MRI techniques that use biophysical models of tissue (Vestergaard-Poulsen et al., 2011, Jespersen et al., 2012, Hansen et al., 2011, Wang et al., 2013) may have higher specificity to changes in tissue cytoarchitecture and the pathological processes of tau accumulation.

NODDI is a recent microstructure imaging technique based on diffusion MRI but using diffusion gradients of different strengths to provide more specific indices of tissue microstructure than DTI (Zhang et al., 2012). NODDI relies on a biophysical model that separates the diffusion of water in brain tissue into three types of microstructural environment: intra-neurite, extra-neurite, and cerebrospinal fluid (CSF) compartments (Zhang et al., 2012). To achieve this, NODDI applies a two level approach by separating the volume fraction of Gaussian isotropic diffusion (IsoVF), representing the freely diffusing water (*i.e.* CSF), from the neural tissue (Zhang et al., 2012). The remaining signal is sub-compartmentalised into components from intra and extra-neurite water, which are non-exchanging. This separation yields important markers: the neurite density index (NDI) (the fraction of tissue that comprises axons or dendrites) and the interdependent extra-neurite volume fraction (the fraction of tissue other than neurites) (Zhang et al., 2012). The dispersion of the neurite structures is further characterised by the orientation dispersion index (ODI) which reflects the spatial configuration of the neurite structures, with large values of ODI corresponding to highly dispersed neurites (*e.g.* grey matter) and small values of ODI to highly aligned axons (*e.g.* white matter tracts) (Zhang et al., 2012).

In this study, we sought to determine the effectiveness of NODDI as an *in vivo* imaging marker to detect tau pathology and demonstrate the improved specificity of the technique over standard DTI measures for detecting AD-like pathology. We report the correlations of both models with immunohistological stains for filamentous tau and evaluate each technique's specificity to tau pathology.

## Methods

Generation of homozygous rTg4510 transgenic mice has been reported previously (Ramsden et al., 2005). Mice were licensed from the Mayo Clinic (Jacksonville Florida, USA) and bred for Eli Lilly by Taconic (Germantown, USA). Mice were imported to the UK for imaging studies at the Centre for Advanced Biomedical Imaging (CABI), London. All studies were carried out in accordance with the United Kingdom's Animals (Scientific Procedures) Act of 1986.

Five TG and five WT female litter matched mice (8.5 months of age) were imaged. The animals were placed in an induction chamber and anesthetised with inhaled isoflurane (2% isoflurane at 1 l/min O_2_) until pedal withdrawal reflex was lost. They were then transferred to an MRI compatible head holder to minimise motion artefacts and maintained at 1.5% isoflurane at 1 l/min O_2_ for the duration of scanning. Core temperature was maintained at 37 °C using a warm air blower feedback system and rectal probe (SA instruments). Physiological monitoring of temperature and respiration was recorded throughout the scan (SA instruments).

The scans were performed on a 9.4T Agilent scanner with Vnmrj 3.1 front end software using the Agilent 205/120HD gradient set. RF transmission was performed with a 72 mm inner diameter volume coil and a 4-channel receiver coil (Rapid Biomedical).

Diffusion-weighted images were acquired using a 4-shot spin echo-planar imaging (EPI) sequence over sixteen slices. The olfactory bulbs were used as an anatomical landmark to maintain consistency in slice positioning between animals and the slices covered the cortex and subcortical structures up to the cerebellum. Imaging parameters were: FoV 20 × 20 mm^2^, resolution 200 × 200 μm, 16 × 500 μm axial slices, TR = 2 s, TE = 24 ms. To enable the NODDI analysis, the diffusion MRI protocol consisted of two shells, which were:

1. Shell one: 30 directions, four b = 0 and b = 2000 s/mm^2^ with parameters: G = 0.349 T/m, Δ = 9.3 ms, δ = 5.5 ms, TR = 2000 ms.
2. Shell two: 20 directions, three b = 0 and b = 1000 s/mm^2^ with parameters: G = 0.250 T/m, Δ = 9.3 ms, δ = 5.5 ms, TR = 2000 ms.

Images were corrected for motion using 2D rigid body registration to a reference volume (first b = 0 image) using DTI-TK (Zhang et al., 2006). Brain masks were created manually with Matlab (Mathworks version 7.1) and the NODDI microstructure parameter maps were estimated from motion-corrected images using the NODDI toolbox (Zhang et al., 2012). The DTI metrics of fractional anisotropy (FA) and mean diffusivity (MD) were generated from the Camino toolbox (Cook et al., 2006) using weighted linear least-squares.

**Supplementary Fig. 1:**
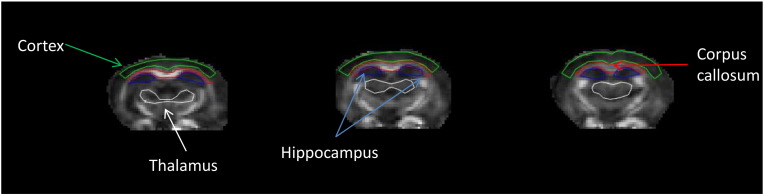
ROI placement on FA maps.

Region of interests (ROIs) were drawn on the three coronal FA maps (Supplementary Fig. 1) which align with anatomical location of histology (− 2 mm from bregma). The four ROIs were the cortex, corpus callosum, hippocampus and thalamus (Fig. 2B); all were manually drawn by the lead author (NC) who was blinded for genotype. These included: Cortex: retrosplenial agranular, retrosplenial granular, motor and somatosensory cortices; Hippocampus: CA1, CA2, CA3 and dentate gyrus; Corpus Callosum: dorsal fornix, corpus callosum, alveus and cingulum; Thalamus: ventral posteromedial nucleus, posterior complex, parafascicular nucleus, lateral posterior nucleus, lateral medial habenula, lateral geniculate complex and intermedial dorsal nucleus. Mean ROI values for FA, MD, ODI, NDI and IsoVF were exported in spreadsheet form for statistical analysis. The intraneurite compartment in healthy grey matter is highly dispersed due to sprawling dendritic processes (Zhang et al., 2012). In this tau pathology mouse model rTG4510 there is a large dendritic degeneration in cortical regions of the grey (Santacruz et al., 2005). This would cause a reduction in dispersion and would be reflected by reduced ODI. The reduction in dendritic volume would also cause a reduction in NDI the volume fraction that comprises the intraneurite compartment. Healthy white matter mainly contains highly orientated axon bundles. In tau pathology, the microtubules of the axon bundles dissociate (Santacruz et al., 2005) which should lead to higher diffusion tortuosity in intraneurite compartment. An increase in diffusion tortuosity would be reflected by an increase in dispersion (higher ODI) and as the white matter region thins a reduction in the neurite density volume (lower NDI). Any increase or decrease in free isotropic diffusion respectively reflects a higher or lower value measured through the tissue volume fraction (Zhang et al., 2012).

The intraneurite compartment in healthy grey matter is highly dispersed due to sprawling dendritic processes (Zhang et al., 2012). In this TG mouse model there is a large dendritic degeneration in cortical regions of the grey matter (Santacruz et al., 2005). This would cause a reduction in dispersion and would be reflected by reduced ODI. The reduction in dendritic volume would also cause a reduction in NDI (the volume fraction that comprises the intraneurite compartment). Healthy white matter mainly contains highly orientated axon bundles. In tau pathology, the microtubules of the axon bundles dissociate (Santacruz et al., 2005) which should lead to higher diffusion tortuosity in intraneurite compartment. An increase in diffusion tortuosity would be reflected by an increase in dispersion (higher ODI) and as the white matter region thins, a reduction in the neurite density volume (lower NDI). Any increase or decrease in free isotropic diffusion respectively reflects a higher or lower value measured through the tissue volume fraction (Zhang et al., 2012).

Directly after *in vivo* imaging the animals are removed from the MRI scanner and terminally anaesthetised with Euthanal (0.1 mL) administered *via* intraperitoneal injection. The thoracic cavities were opened and the animals perfused through the left ventricle of the heart with 15–20 mL of saline (0.9%) followed by 50 mL of buffered formal saline at a flow rate of 3 mL per minute. Following perfusion, the animal was decapitated, defleshed and the lower jaw removed. All brains were stored in-skull in buffered formal saline at 4 °C before being dispatched for histology.

Brain samples were then processed using the Tissue TEK® VIP processor (GMI Inc, MN USA). After processing, sections were embedded in paraffin wax to allow coronal brain sections to be cut. Serial sections (6–8 μm) were taken using HM 200 and HM 355 (Thermo Scientific Microm, Germany) rotary microtomes.

Immunohistochemistry (IHC) was performed using a primary antibody for tau phosphorylated at serine 409 (PG-5; 1:500 from Peter Davies, Albert Einstein College of Medicine, NY, USA). Secondary antibody was applied and slides were then incubated with avidin biotin complex (ABC) reagent for 5 min (M.O.M. kit PK-2200, Elite ABC rabbit kit PK-6101, or Elite RTU ABC PK-7100 Vector Labs). After rinsing, slides were treated with the chromogen 3, 3′-diaminobenzidine (DAB; Vector Laboratories, SK-4100) to allow visualisation. The slides were then cover slipped, dried and digitised using an Aperio Scanscope XT (Aperio Technologies Inc., CA, USA).

Images were viewed and analysed with Aperio ImageScope software (version 10.2.2319). In this study, two sections that align with the diffusion slices for each mouse were analysed. For each section stained, regions of specific interest (in this case the cortex, corpus callosum, hippocampus and thalamus) were delineated. The density of PG-5 immunoreactivity was quantified within these regions of interest using Aperio ImageScope and exported into spreadsheets for statistical comparison with diffusion measurements.

The t-test of group differences between WT and TG and Pearson correlations of diffusion metrics with PG-5 immunoreactivity were carried out using Graphpad Prism (version 5) (Motulsky et al., 1994). Adjustment for multiple comparisons was performed for each model using false discovery rate (FDR) correction and the level of significance was set at 0.05.

## Results

**Fig. 1:**
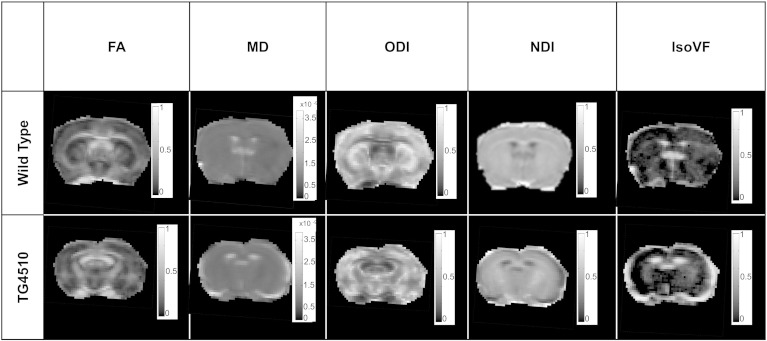
Representative coronal FA, MD (× 10^− 9^ m^2^/s), ODI, NDI and IsoVF maps at − 2 mm from bregma.

Representative maps of fractional anisotropy (FA), mean diffusivity (MD), orientation dispersion (ODI), neurite density (NDI), and isotropic diffusion (IsoVF) for WT and TG mice are presented in Fig. 1. The FA and ODI maps show the highly anisotropic nature of the corpus callosum white matter. The IsoVF map clearly delineates CSF and the associated ventricular enlargement in the TG.

**Fig. 2:**
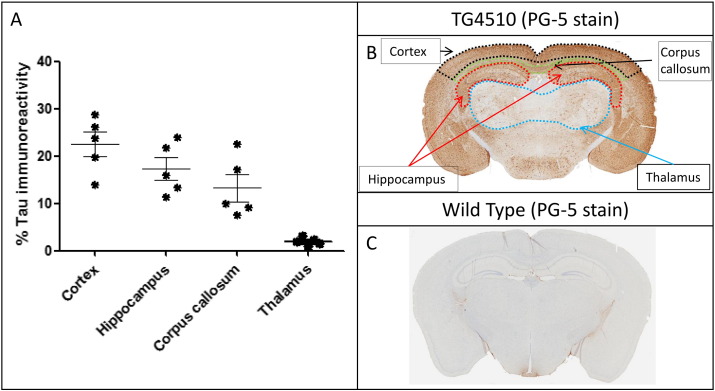
Percentage tau burden measured for each animal based on distinct anatomical regions (A) and representative brain maps of tau immunohistology and regions of interest in TG4510 (B) and wildtype (C) animals.

The output of the ROI analysis of the four regions selected: three grey matter regions (cortex, hippocampus and thalamus) and one white (corpus callosum) (Fig. 2B), were compared with the percentage tau burden measured through immunohistology in the same four regions. The cortex and the hippocampus, as expected, presented with the highest degree of tau burden and the thalamus had the lowest tau burden (Fig. 2A).

### Cortex

**Fig. 3:**
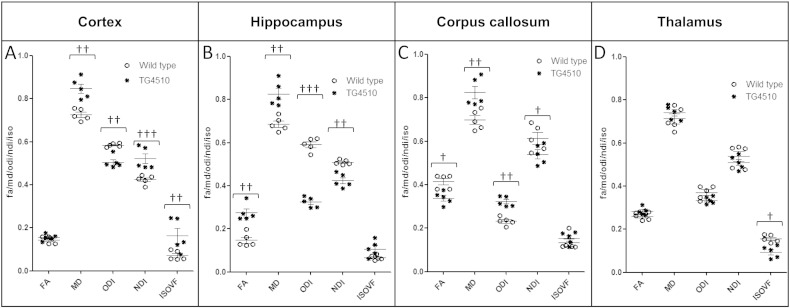
ROI quantification of FA, MD (× 10^−9^ m^2^/s), ODI, NDI and IsoVF for each animal based on distinct anatomical regions. († = p < 0.05, †† = p < 0.01, ††† = p < 0.001).

**Fig. 4:**
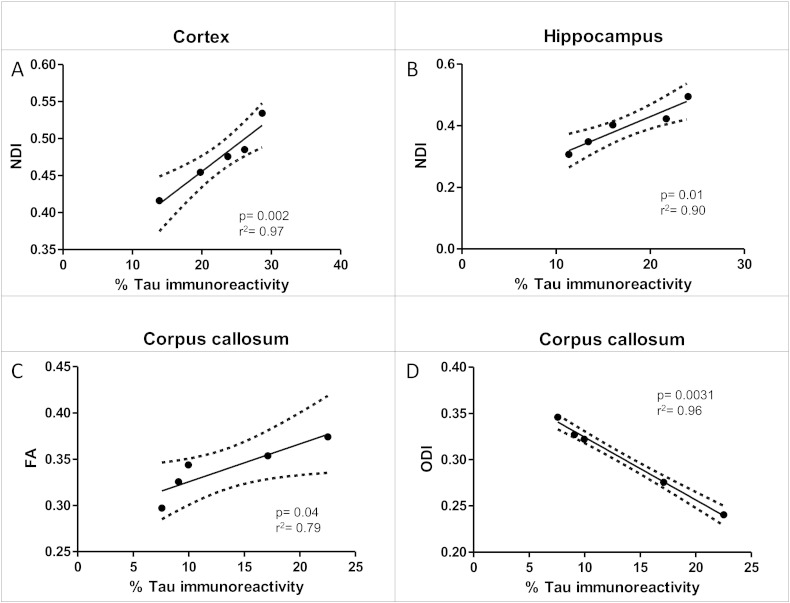
Correlation of significant dMRI measurements with percentage tau burden.

In the cortex (region with the highest tau burden, Fig. 2A), all NODDI parameters distinguished the TG group from the WT group. The TG group had lower ODI (Tratio = 3.8, df = 8, p < 0.01), higher NDI (Tratio = 4.7, df = 8, p < 0.001) and higher IsoVF (Tratio = 3.6, df = 8; p < 0.01) in the TG group (Fig. 3A). There was also a significant higher MD (Tratio = 3.9, df = 8, p < 0.01) (Fig. 3A) in the TG group. However, only neurite density (NDI) correlated significantly with the degree of tau burden (p < 0.002) (Fig. 4A).

### Hippocampus

In the hippocampus (region with the second highest tau burden, Fig. 2A), there was a significantly higher FA (Tratio = 4.7; df = 8; p < 0.01) and MD (Tratio = 6.6; df = 8; p < 0.01), as well as lower ODI (Tratio = 12.6; df = 8; p < 0.0001) and NDI (Tratio = 5.9; df = 8; p < 0.01) in the TG group, with no separation between groups in the IsoVF (Fig. 3B). As in the cortex, NDI was the only parameter which correlated with the percentage of tau burden (p < 0.01) (Fig. 4B).

### Thalamus

In the thalamus (region with the lowest tau burden, Fig. 2A), FA, MD, ODI and NDI could not discriminate between WT and TG groups. However there was a significantly lower isotropic diffusion volume fraction (IsoVF) in the TG mice compared with WT animals (Tratio = 2.9; df = 8; p < 0.05) (Fig. 3D).

### Corpus callosum

In the corpus callosum ROI, there was greater tau staining observed than in the thalamus but less than in the hippocampus and the cortex (Fig. 2A). In the TG group, there was lower anisotropy (FA, Tratio = 3.9; df = 8; p < 0.05) and higher dispersion (ODI, Tratio = 9.6; df = 8; p < 0.01) and diffusivity (MD, Tratio = 5.1; df = 8; p < 0.01), with a lower neurite density (NDI, Tratio = 3.6; df = 8; p < 0.05) in the TG group (Fig. 3C). In this white matter region both FA (R^2^ = 0.79 and p = 0.04) and ODI (R^2^ = 0.96 and p = 0.003) correlated with tau burden (Fig. 4C & D), however ODI demonstrated a far stronger correlation (R^2^ = 0.96 and p = 0.003).

## Discussion

In this work, we have shown that NODDI indices correlated with histological measurements of tau pathology in grey matter regions in a mouse model of human tauopathy whereas traditional DTI indices of MD and FA do not.

**Supplementary Fig. 2:**
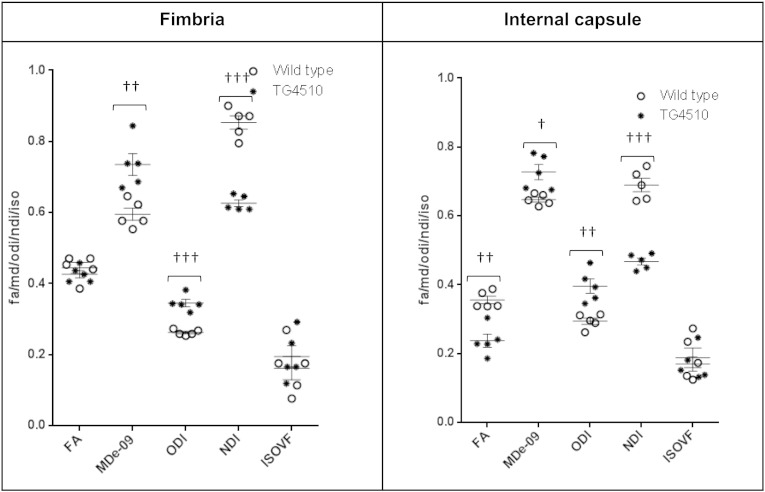
ROI quantification of FA, MD (× 10^− 9^ m^2^/s), ODI, NDI and IsoVF for each animal based on distinct anatomical regions († = p < 0.05, †† = p < 0.01, ††† = p < 0.001).

The significantly lower FA in the white matter tract of the corpus callosum (CC) is in good agreement with previous studies of the TG animals at 8 months (Sahara et al., 2014, Wells et al., in press). Lower FA and higher MD in the CC have been reported in AD patients when compared with healthy volunteers (Bozzali et al., 2001) and more recent studies have found that reductions in the white matter FA in AD patients correlate with CSF AD biomarkers of total and phosphorylated tau (Amlien et al., 2013). The higher ODI and reduced FA in the white matter reflect the previously reported disorganisation of the white matter due to elevated tau burden (Sahara et al., 2014). The ability of the NODDI technique to separate the microstructural compartments that all contribute to DTI metrics is reflected by the higher sensitivity of ODI reduced directionality of white matter tissue due to tau pathology (Sahara et al., 2014) (Supplementary Table 1). Furthermore we examined two additional regions of the white matter, the fimbria and internal capsule. In these two regions we found similar changes to that of the corpus callosum in the TG group with higher ODI, reduced NDI and no significant difference in IsoVF in the NODDI technique (Supplementary Fig. 2).

In the cortex, a grey matter region which is normally characterised by sprawling dendritic processes, the dispersion (*i.e.* ODI) was reduced in the TG, and the neurite density (NDI) and isotropic diffusion (IsoVF) were higher. The increase in filamentous tau burden (Fig. 2A) and intraneuronal inclusions has resulted in cortical thinning (Fig. 2B). Previous findings have reported a loss of pyramidal cells, a reduction in cortical volume and an increase in neurite density at this time point in this TG animal model (Ludvigson et al., 2011). Lower ODI and higher NDI in the TG animals implies a similar reduction in dendritic complexity, due to atrophy of the cortical layers and accompanied loss of neurite structures as previously reported in this animal model (Ludvigson et al., 2011). As such the tissue would become less dispersed; leading to lower ODI and the reduced volume of cortex with higher density is reflected by higher NDI in the TG group. As the cortical structure atrophies the space becomes occupied by CSF. The higher CSF contamination increases the free diffusion compartment reflected by a higher isotropic volume fraction (IsoVF). The higher MD in this region is likely due to the partial volume effects of the CSF contamination, and NODDI's ability to remove the effect of CSF contamination increases the specificity to the cytoarchitecture. Interestingly, NDI was the only parameter to correlate with degree of tau pathology, indicating that this parameter may be sensitive to the underlying nature of the disease process. However two of the animals may be driving the significant differences in the isotropic volume fraction and neurite density observed in the cortex. Similarly in the hippocampus, the neurite density was the only parameter that correlated with histological measures of tau burden. There are also region-specific effects of tau pathology in the marked atrophy of hippocampus with previously reported neurite loss at 8.5 months amounting to 85% in dentate gyrus, 82% in CA1 and 69% in CA2/3 (Spires et al., 2006). Currently we do not have the resolution in dMRI data to separate each compartment of the hippocampus, however the dramatic loss in neurite projections is reflected by the reduced NDI, and the reduced dispersal of neuron projections is reflected by a lower ODI. The correlations in both the cortex and hippocampus indicate that NDI may provide higher specificity to tau pathology formation in grey matter relative to MD or FA. Correspondingly, in the thalamus, which has a very low degree of tau burden in comparison to the hippocampus and cortex, the FA, MD, ODI and NDI did not discriminate TG from WT, although the IsoVF was lower in the TG group. However no correlation was found between IsoVF and tau burden in the thalamus (Supplementary Table 1). Previous studies have reported a significant higher MD in the thalamus at 8.5 months in this animal model (Wells et al., 2015). However due to the lower numbers in this study this change may not be detected.

DTI has great potential value as a clinical biomarker in AD (Oishi et al., 2011, Keihaninejad et al., 2013, Ryan et al., 2013). However, although attempts have been made (Molinuevo et al., 2014, Patil and Ramakrishnan, 2014), it is difficult to relate the parameters directly to pathological or biological processes. In contrast, a NODDI parameter derived from a biological model of brain cytoarchitecture has displayed a close correlation with the degree of tau pathology in the TG mouse model (Supplementary Table 1). This approach could extend the applicability of diffusion imaging in both preclinical models and clinical AD by providing a window on grey matter microstructural alterations and their relationship to underlying disease processes, other biomarkers of AD pathology and clinical or behavioural phenotype.

## Limitations

Further work is required to apply this technique to a multi-parameter longitudinal study over the time course of disease. This would determine the sensitivity of the NODDI metrics to other novel and established MR methods in the pre-symptomatic phase of the disease. We envisage that a multi-parameter longitudinal study would have three separate animal groups of (1) WT littermates; (2) untreated TG and (3) doxycycline treated TG group (which suppresses tau expression) investigated from 2 months to 8 months. This study would aim to determine the specificity of the NODDI metrics to pathology in the pre-symptomatic phase of the disease and during controlled expression. Although we have demonstrated specificity of NODDI metrics in this tau model the number of mice in this study is low and greater conspicuity may be reached by increasing the sample size.

## Conclusion

The results of this cross-sectional study suggest that NODDI measurements could provide a higher degree of specificity to the pathological effects of tau in grey matter, and higher interpretability with respect to the underlying biological processes, in comparison to traditional DTI measures.

The NODDI metrics discriminated between the TG and WT groups, and the correlations between the immunohistological measures of pathological tau and NODDI measures in the cortex, hippocampus and corpus callosum demonstrated the heightened specificity of this technique in comparison to DTI (Supplementary Table 1). The correlation of microstructural grey and white matter changes with pathology represents a new target of investigation in AD which may serve as an early diagnostic marker of pathology, however in light of the limitations outlined above these result must be considered as exploratory at this point.

The following are the supplementary related to this article.

**Supplementary Table 1:**
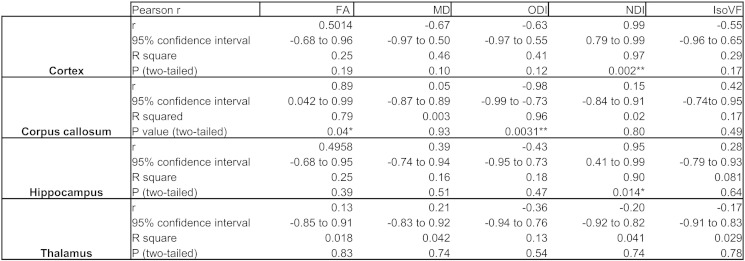
Correlations of dMRI measures with immunohistological percentage tau burden.
